# Identification of four novel large deletions and complex variants in the α-globin locus in Chinese population

**DOI:** 10.1186/s40246-023-00486-4

**Published:** 2023-04-25

**Authors:** Xiuqin Bao, Jicheng Wang, Danqing Qin, Cuize Yao, Jie Liang, Kailing Liang, Yukun Zeng, Li Du

**Affiliations:** 1 Medical Genetics Center, Guangdong Women and Children Hospital, Xingnan Road 521, Guangzhou, 510010 Guangdong People’s Republic of China; 2 Maternal and Children Metabolic-Genetic Key Laboratory, Guangdong Women and Children Hospital, Guangzhou, 510010 Guangdong People’s Republic of China; 3 Thalassemia Diagnosis Center, Guangdong Women and Children Hospital, Guangzhou, 510010 Guangdong People’s Republic of China

**Keywords:** α-Globin locus, Large deletions, Microcytic hypochromic anemia, Rare and complex variants, Single-molecular real-time sequencing

## Abstract

**Background:**

At present, the methods generally used to detect α-thalassemia mutations are confined to detecting common mutations, which may lead to misdiagnosis or missed diagnosis. The single-molecule real-time (SMRT) sequencing enables long-read single-molecule sequencing with high detection accuracy, and long-length DNA chain reads in high-fidelity read mode. This study aimed to identify novel large deletions and complex variants in the α-globin locus in Chinese population.

**Methods:**

We used SMRT sequencing to detect rare and complex variants in the α-globin locus in four individuals whose hematological data indicated microcytic hypochromic anemia. However, the conventional thalassemia detection result was negative. Multiplex ligation-dependent probe amplification and droplet digital polymerase chain reaction were used to confirm SMRT sequencing results.

**Results:**

Four novel large deletions were observed ranging from 23 to 81 kb in the α-globin locus. One patient also had a duplication of upstream of *HBZ* in the deletional region, while another, with a 27.31-kb deletion on chromosome 16 (hg 38), had abnormal hemoglobin Siriraj (Hb Siriraj).

**Conclusion:**

We first identified the four novel deletions in the α-globin locus using SMRT sequencing. Considering that the conventional methods might lead to misdiagnosis or missed diagnosis, SMRT sequencing proved to be an excellent method to discover rare and complex variants in thalassemia, especially in prenatal diagnosis.

## Introduction

As one of the most common inherited disorders globally [1], α-thalassemia mainly results from a deletion in the α-globin cluster, located on chromosome 16. It mainly occurs in tropical and subtropical regions, including southern China, with a high carrier rate of 11.31% and 17.55%, in Guangdong and Guangxi provinces, respectively [2, 3]. The most common α-thalassemia mutation is -α^3.7^ (5.32%), followed by --^SEA^ (4.82%) and -α^4.2^ (3.23%), in southern China [4]. When an individual is homozygous for --^SEA^ deletion, it results in Hb Bart’s hydrops fetalis and leads to death during gestation or several hours after birth. Conversely, when an individual is heterozygous for --^SEA^ deletion compounded with -α^3.7^ or -α^4.2^ deletion, it causes HbH disease. Hence, the patient may depend on regular blood transfusions to survive [5]. Large deletion in the α-globin locus usually results in significant manifestations, with lower mean corpuscular volume (MCV) and mean corpuscular hemoglobin (MCH) levels than the heterozygous with deletional α-thalassemia --α^3.7^ or -α^4.2^. Therefore, identifying the rare large deletional mutation in the α-globin locus is important in clinical diagnosis, especially in prenatal diagnosis.

At present, the conventional methods to detect large deletional mutation in α-globin cluster include Multiplex ligation-dependent probe amplification (MLPA) [6], gap-Polymerase Chain Reaction (gap-PCR) [7] and droplet digital polymerase chain reaction (ddPCR) [7–9]. However, these methods have several limitations. MLPA is mainly used for the detection and genotype confirmation of copy number variants (CNVs) of unknown types in individual cases because of its complicated, time-consuming, and costly operations. Further, MLPA cannot determine the precise breakpoint of the deletion. Although multiplex gap-PCR is relatively less expensive and does not require high-end equipment, it can only detect the known common deletional mutations, inevitably leading to a missed diagnosis. Although ddPCR can accurately detect CNVs, it cannot characterize the precise breakpoint of the deletion. With the rapid development of sequencing techniques, third-generation sequencing [10], also known as single-molecule real-time (SMRT) sequencing, is being used to identify common and rare variants in thalassemia [11, 12]. Compared with the conventional methods, SMRT sequencing, with the advantages of high throughput and comprehensive site coverage, can accurately identify duplications or breakpoints of deletions via only a single experiment. It provides direct evidence for diagnosing complex structural variations of α-thalassemia. Yan et al. [12] suggested that the conventional methods used to detect α-thalassemia had a rate of missed diagnosis of up to 4.17%, while SMRT sequencing could achieve 100% precise detection results. Further, the results of SMRT sequencing were assessed using the genomic visualization tool integrative genomics viewer (IGV). In particular, the tool provided a platform to easily read the SMRT sequencing and helped researchers to increase their understanding of variations of α-thalassemia. In this study, we recruited four patients whose hematological data indicated microcytic hypochromic anemia. Suspension array system and Sanger sequencing were used to detect the α- and β-thalassemia mutations, while MLPA and ddPCR were used to detect the copy number variants in α-globin cluster. Finally, we used SMRT sequencing to identify the rare and complex variants in the α-globin locus and found four different novel deletions ranging from 23 to 81 kb.

## Materials and methods

### Samples and hematological analysis

Subject 1 was from Guangxi Province, whereas subjects 2–4 were from Guangdong Province, China. The routine blood test revealed that all patients had microcytic hypochromic anemia. However, the thalassemia gene test result was negative. Therefore, they came to our center for further diagnosis. All subjects provided written informed consent.

Fresh peripheral blood (PB) sample was collected, and the hematological parameters were analyzed using a Sysmex XN5000 automated hematology analyzer (Sysmex Corporation, Kobe, Japan). Hb quantification was performed using an automated capillary electrophoresis system (CE) (Sebia Capillarys 2, France).

### Common α- and β-thalassemia detection

Common α-globin [-α^3.7^ (rightward), -α^4.2^ (leftward), --^SEA^ (Southeast Asian), Hb Constant Spring (Hb CS or *HBA2*: c.427T > C), Hb Quong Sze (Hb QS or *HBA2*: c.377  > C) and Hb Westmead or *HBA2*: c.369C > G], and β-globin [codons 41/42 (–TTCT) (*HBB*: c.126_127delCTTT), IVS-II-654 (C > T) (*HBB*: c.316-197C > T) –28 (A > G) (*HBB*: c.-78A > G), codons 71/72 (+ A) (*HBB*: c.216_217insA), codon 17 (*A*AG > *T*AG) (*HBB*: c.52A > T), codon 26 (*G*AG > *A*AG) (Hb E or *HBB*: c.79G > A), codon 31 (–C) (*HBB*: c.94delC), codons 27/28 (+ C) (*HBB*: c.84_85insC), IVS-I-1 (G > T) (*HBB*: c.92 + 1(G > T), codon 43 (*G*AG > *T*AG) (*HBB*: c.130G > T), –32 (C > A) (*HBB*: c.-82 > A), –29 (A > G) (*HBB*: c.-79A > G), –30 (T > C) (*HBB*: c.-80T > C), codons 14/15 (+ G) (*HBB*: c.45_46insG), Cap + 40–43 (–AAACA) (*HBB*: c.-11_-8delAAACA), initiation codon (A*T*G > A*G*G) (*HBB*: c.2T > G) and IVS-I-5 (G > C) (*HBB*: c.92 + 5G > C)] mutations were detected using the suspension array system.

### Multiplex ligation-dependent probe amplification (MLPA)

MLPA was performed using SALSA MLPA Probemix P140-C1 HBA (MRC-Holland, Amsterdam, the Netherlands) following the manufacturer’s protocols. In brief, DNA was diluted to 28–30 ng/µL, and 4 µL of DNA was added to each tube and placed in a thermocycler. The thermocycler program was started at 98 °C for 20 min, and then paused at 25 °C. We ensured that the samples were placed at 25 °C before removing the tubes from the thermocycler. Then, 0.75 µL MLPA buffer and 0.75 µL probe mix were added and initially incubated for 1 min at 95 °C and then for a further 16 h at 60 °C, after which the mixture was stored at 54 °C. Subsequently, 12.5 µL ultrapure water, 1.5 µL of ligase buffer A, 3 µL of ligase buffer B, and 0.5 µL of Ligase-65 enzyme were added. The mixture was initially denatured for 15 min at 54 °C and then a further 5 min at 98 °C, after which the mixture was stored at 20 °C. PCR was performed as follows: 3.75 µL of ultrapure water, 1 µL of SALSA PCR primer mix, and 0.25 µL of polymerase master mix were added and incubated in the thermocycler for 35 cycles at 95 °C for 30 s, 60 °C for 20 s, and 72 °C for 60 s. After 35 cycles, DNA was incubated at 72 °C for 20 min and stored at 15 °C. The PCR products were analyzed using the GenomeLab GeXP Genetics Analysis System (BeckMan). Ratio < 0.7 was defined as deletion, while ratio > 1.3 was defined as duplication.

### Droplet digital polymerase chain reaction (ddPCR)

We used ddPCR to calculate the copy number of *HBA1* and *HBA2*. ddPCR was performed as previously described [8]. We used *RPP30* to calculate the copy number, which had two copy numbers of *RPP30* gene, as the reference gene. The copy number was calculated as follows: CNV = (*X*/*Y*)*N*_ref_, where X = the concentration (copies/μl) of target genes, Y = the concentration (copies/μl) of *RPP30*, and *N*_ref_ = the copy number of *RPP30* (usually 2).

### SMRT sequencing and Sanger sequencing

Experiments were conducted by Berry Genomic Corporation (Beijing, China) as described in a previous study [13]. Briefly, genomic DNA was subjected to PCR with primers covering the majority of known structural variations, single nucleotide variants (SNVs) and indels in the *HBA1*, *HBA2* and *HBB* regions. PCR products were ligated with barcoded adaptors by a one-step end-repair and ligation reaction to construct pre-libraries, which were pooled together by equal mass and converted into an SMRT dumbbell (SMRTbell) library using the Sequel Binding and Internal Ctrl Kit 3.0 (Pacific Biosciences). Then, the SMRTbell library was sequenced under circular consensus sequencing (CCS) mode on the Sequel II platform (Pacific Biosciences). After sequencing, raw subreads were converted into CCS reads, debarcoded and aligned to genome assembly hg38 in SMRT Link software (Pacific Biosciences). Structural variations were identified based on HbVar, Ithanet and LOVD databases, and SNVs and indels were identified using FreeBayes1.3.4 (https://www.geneious.com/plugins/freebayes; Biomatters, Inc., CA, USA). In subject 1, 3, and 4, the DNA region encompassing the duplication and deletion probes by MLPA was amplified by specifically designed primers and sequenced on the Sequel II platform. The converted CCS reads were aligned to hg38, and the precise regions of duplication and deletion were determined.

Sanger sequencing was used to detect the rare α- or β-thalassemia mutations. We used the following primers to amplify the *HBB* gene (F: 5′-AGAAGATATGCTTAGAACC-3′, R: 5′ TTGCTATTGCCTTAACCCAGAA-3′) and *HBA* gene (*HBA1*, F: 5′-TGGAGGGTGGAGACGTCCTG-3′, R: 5′-TCCATCCCCTCCTCCCGCCCCTGCCTTTTC-3′; *HBA2*, F: 5′-GATGGGCGGGAGTGGAGT-3′, R: 5′-GGACAGGGGATGGTTCAGC-3′). The products were then sequenced.

## Results

**Fig. 1:**
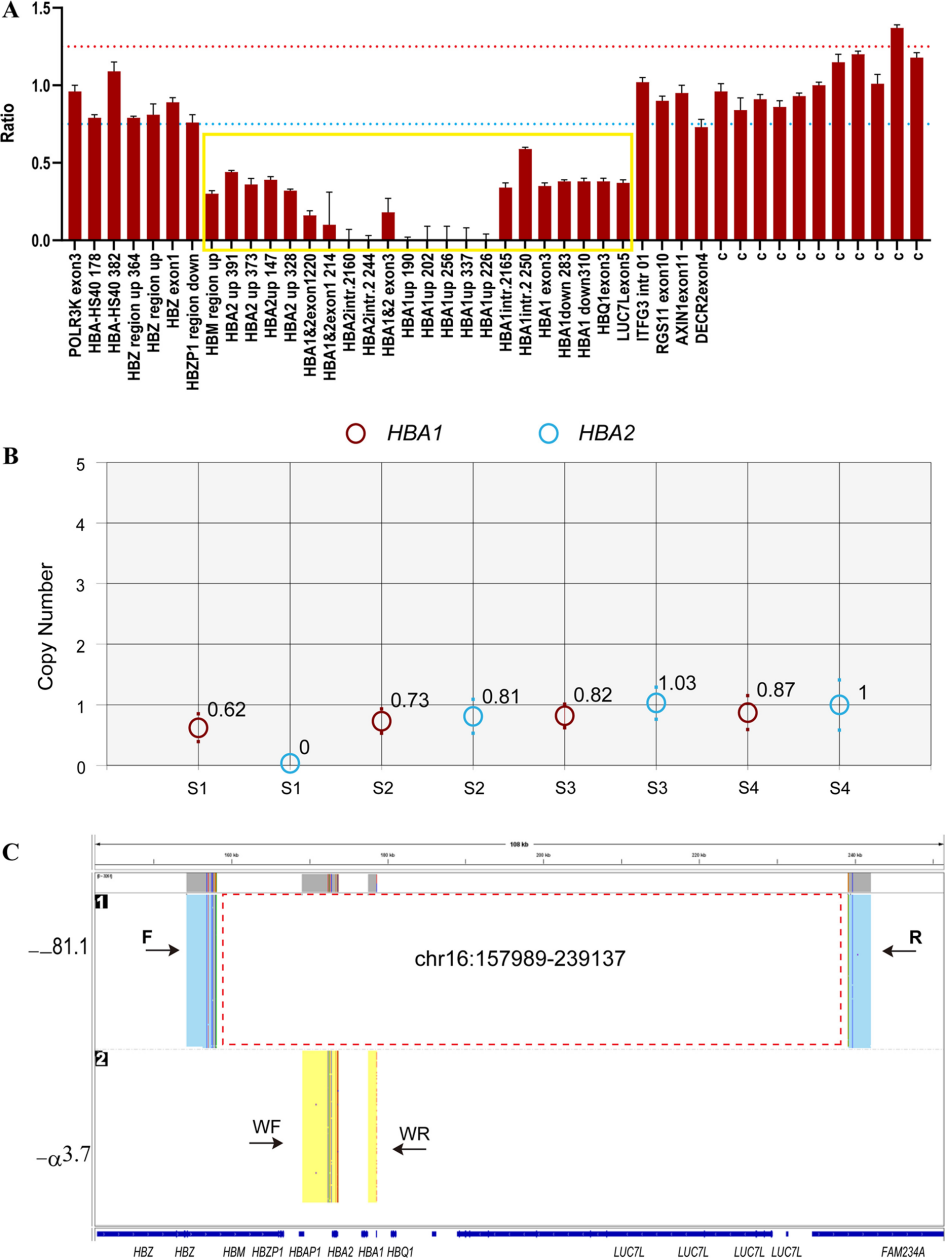
Deletional analysis of subject 1. **A** MLPA analysis in the α-globin cluster. The yellow box indicates the deletional region. The red dashed line shows a ratio of 1.25, while the blue dashed line shows a ratio of 0.75. **B** ddPCR results of subjects. S1 to S4 indicate subject 1 to 4. Copy numbers > 0.6 and < 1.25 were considered one copy. **C** SMRT sequencing analysis in the α-globin cluster. The light yellow and blue regions indicate the two alleles of the α-globin gene cluster. The red dashed box shows the deletional region. Blue boxes indicate the relative positions of the genes on chromosome 16. The vertical colored lines indicate nucleotides A (green), T (red), C (blue) and G (orange) discordant with alignment to the hg38 reference sequence. F, Forward primer; R, reverse primer. We used other primers (such as WF and WR) to amplify the wild-type allele because the fragment was up to 40 kb in the wild type allele, which PCR was unable to amplify

Subject 1 was diagnosed with -α^3.7^ homozygotes with the detection of common α- and β-thalassemia mutations using the suspension array system. However, her hematological data showed microcytosis and hypochromic red cells, with Hb, MCV and MCH of 81 g/L, 67 fL and 19 pg, respectively (Table 1). These values were much higher than those found in individual homozygous for -α^3.7^. In addition, the hemoglobin quantification showed that she had 6.3% HbH and 1.1% HbA2, which contradicted the result of gene test. We performed MLPA to detect the rare deletional mutation so as to explain the obviously inconsistent result. We found a large deletion ranging from upstream of the *HBM* region to the exon of *LUC7L* (Fig. 1A) within the α-globin cluster. The result of ddPCR also indicated one copy in the *HBA1* region and no copy in *HBA2* region (Fig. 1B). We further performed SMRT sequencing to precisely determine the breakpoint of this deletion. The SMRT sequencing analysis recapitulated the MLPA result and indicated an 81.1-kb deletion extending from downstream of *HBZ* to the first intron of the *FAM234A* gene. The precise position of the deletion was located between 157,989 and − 239,137 in chromosome 16 (NC_000016.10:g.157989_239137del, GRch38/hg38) (Fig. 1C).

**Table 1:** Phenotype and genotype data of the four individuals

Patient	Gender (F/M)	Age (year)	Hb (g/L)	MCV (fL)	MCH (pg)	HbA2 (%)	HbH/ abnormal Hb	HBA genotype^#^	HBB genotype
Subject 1	F	26	81	67.0	19.0	1.1	6.3% HbH	-α^3.7^/--^81.1^	N/N
Subject 2	F	35	100	66.0	21.0	4.3	8.8% Hb Siriraj	--^27.31^ /αα	c.22G > A/N
Subject 3	M	28	137	66.6	20.3	2.2	-^*^	--^45.2^ /αα	N/N
Subject 4	M	28	140	66.9	22.4	2.3	-^*^	--^23.1^/αα	N/N

^#^The genotype was named based on the deletion size

*Indicated normal

*N* Normal

**Fig. 2:**
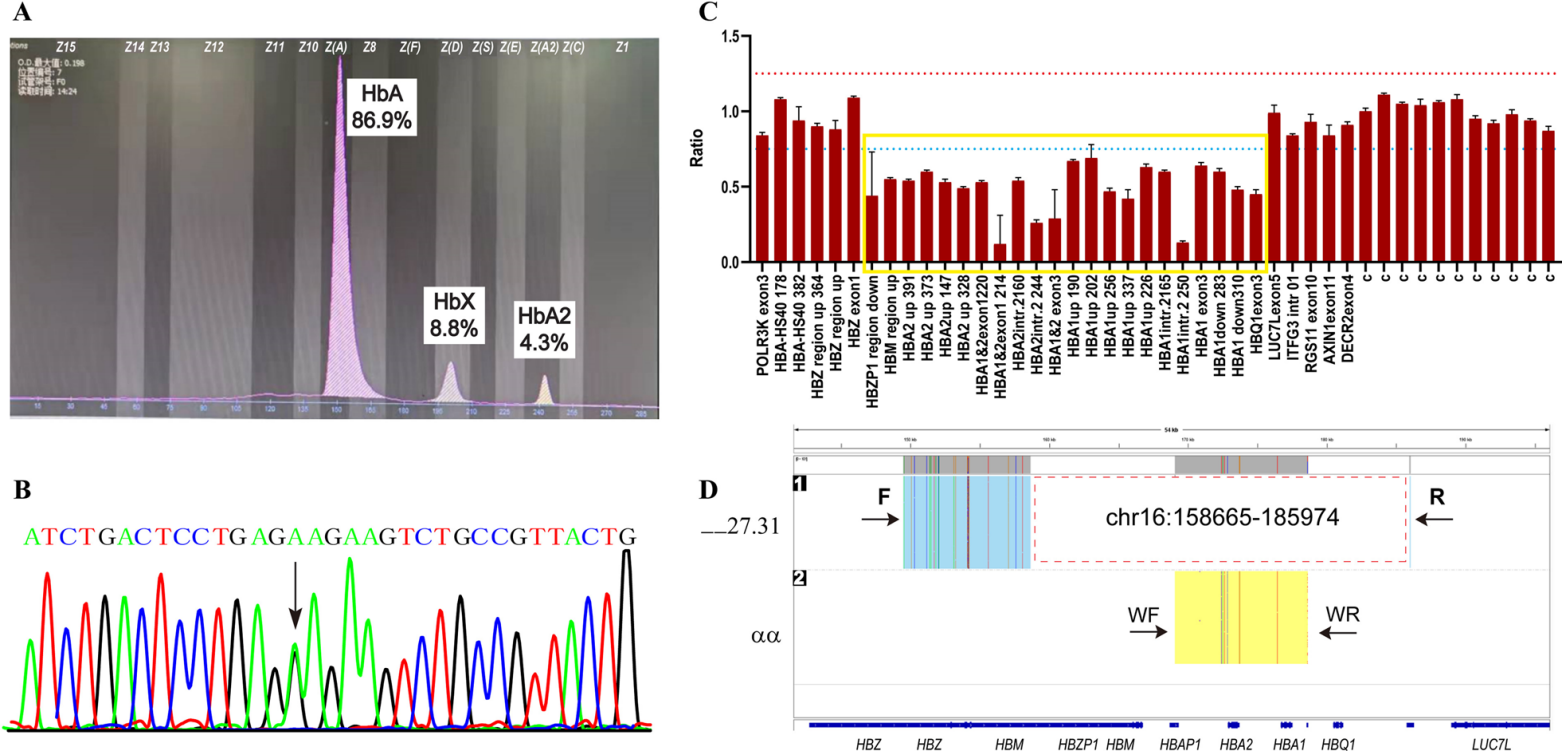
Deletional analysis of subject 2. **A** Hematological analysis of subject 2. **B** Sanger sequencing in the *HBB* gene. The arrow shows the heterozygous mutation in *HBB*. **C** MLPA analysis in α-globin cluster. The yellow box indicates the deletional region. The red dashed line shows a ratio of 1.25, while the blue dashed line shows a ratio of 0.75. **D** SMRT sequencing analysis in α-globin cluster. The red dashed box shows deletional region. F, forward primer; R, reverse primer

The result of common α- and β-thalassemia mutations detection was negative in subject 2. However, she has an increased HbA2 level (4.3%); therefore, we performed sanger sequencing and found that she was heterozygous for abnormal Hb G-Siriraj (β7 Glu > Lys, *HBB*:c.22G > A) (Fig. 2A, B), which was confirmed by SMRT sequencing. However, her Hb, MCV and MCH levels were 81 g/L, 66 fL and 21 pg, respectively (Table 1), which contradicted the manifestation in people carrying Hb G-Siriraj, who behave normally. MLPA was performed to detect a rare deletion in the α-globin cluster. We observed a large heterozygous deletion ranging from *HBM* to *HBQ1* (Fig. 2C). ddPCR also showed one copy for both *HBA1* and *HBA2* (Fig. 1B). We used SMRT sequencing and found a 27.31-kb deletion extending from downstream of *HBZ* to downstream of *HBQ1* to characterize the breakpoint of the deletion (Fig. 2D). The precise breakpoint was located between 158,665 and − 185,974 in chromosome 16 (NC_000016.10:g. 158665_185974del, GRch38/hg38).

**Fig. 3:**
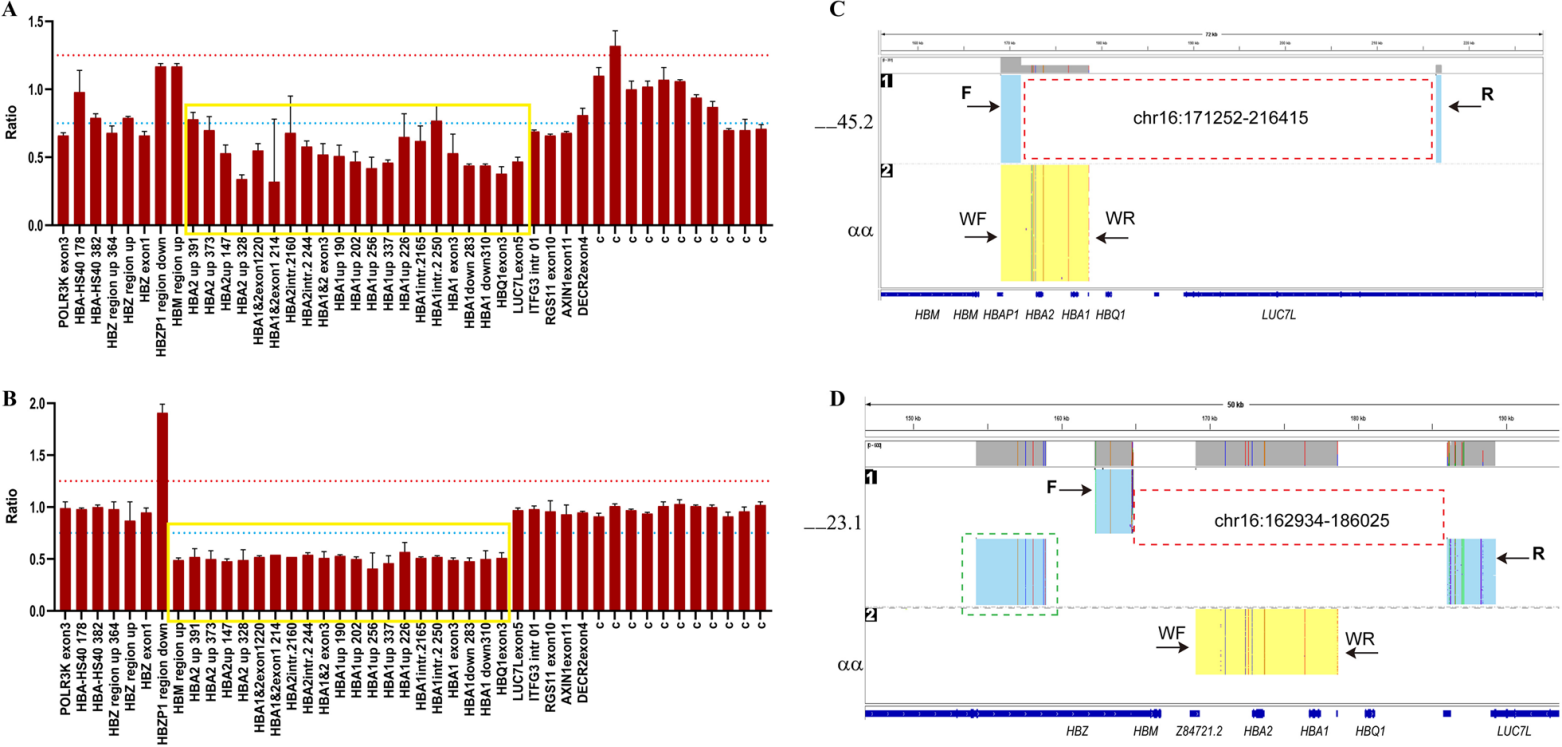
Deletional analysis of subject 3 and 4. **A** and **B** MLPA analysis results in subject 3 (**A**) and subject 4 (**B**). The yellow box indicates the deletional region. The red dashed line shows a ratio of 1.25, while the blue dashed line shows a ratio of 0.75. **C** and **D** SMRT sequencing analysis in the α-globin cluster in subject 3 (**C**) and subject 4 (**D**). The red dashed box shows the deletional region. The green dashed box indicates the duplicated fragment. F, Forward primer. R, reverse primer

Subject 3 and 4 both were male from Huizhou, Guangdong Province, China. Common thalassemia mutations were not found in both patients. Further, rare deletional α-thalassemia mutations in the Chinese population including --^THAI^, -α^27.6^ and -α^21.9^, were also not found in subject 3 and 4. However, their hematological data (Table 1) indicated that they might have rare variants in the α-globin locus. Giving that their wives carry the α-thalassemia mutations --/-α^3.7^ and --/αα, respectively, further studies should be performed to avoid giving birth to babies with major α-thalassemia. MLPA and SMRT sequencing were carried out to characterize the rare variants. The MLPA analysis showed that subject 3 had a heterozygous deletion ranging from upstream of *HBA2* to the exon 5 of *LUC7L* (Fig. 3A), while subject 4 had a deletion from the upstream of *HBM* to exon 3 of *HBQ1* (Fig. 3B). Moreover, the result of ddPCR displayed one copy in both *HBA1* and *HBA2* in subject 3 and 4 (Fig. 1B). Based on the result of SMRT sequencing, the precise breakpoint in subject 3 was located between 171,252 and − 216,415 in chromosome 16 (NC_000016.10:g. 171252_216415del, GRch38/hg38), creating a 45.2-kb deletion (Fig. 3C). In subject 4, a complex variant included deletion and duplication. The deletion extended from 162,934 to 186,025 in chromosome 16 (NC_000016.10:g. 162934_186025del, GRch38/hg38), creating a 23.1-kb deletion. Meanwhile, a duplicated fragment ranging from 153,060 to 158,979 was inserted in the deletional region (Fig. 3D). These novel variants were submitted to the database dbVar with the accession number nstd221.

## Discussion

The precise breakpoints of many large deletions in the α-globin gene cluster have not been identified [14] due to technical limitations. At present, with the rapid development of sequencing techniques, SMRT sequencing can easily characterize the genotype and breakpoint. SMRT sequencing can detect α- and β-thalassemia genes without interrupting the DNA and directly read the full-length (up to 30–100 kb) gene sequence [15]. This means that SMRT sequencing can detect all the reported hotspots and rare variant sites and their arrangements in thalassemia. Further, SMRT sequencing can facilitate the simultaneous detection of α- and β-thalassemia. According to a recent study [12], SMRT sequencing detected 28 more types of variants compared with conventional technologies (including gap-PCR, PCR-reverse dot blot (RDB), suspension array system and MLPA). The positive detection rate using SMRT sequencing was 9.91% higher than that of conventional technology, and SMRT sequencing increased the detection of thalassemia genes. The comprehensive analysis of thalassemia alleles organized by Central South University [13] for identifying both α and β thalassemia genetic carrier status using SMRT sequencing showed that the SMRT sequencing could detect 33 more types of negative variants compared with standard thalassemia variant PCR panel testing. In the prenatal diagnosis, it is important to characterize the genotype of one partner in the couple if the other carries a thalassemia mutation, as the fetus has a rate of 25% chance of having major thalassemia. If someone is heterozygous for --^SEA^/, their partner, who actually is heterozygous for large deletions including *HBA1* and *HBA2* in α-globin cluster, is found to be negative via conventional thalassemia gene testing. This missed diagnosis by conventional detection methods may result in 25% of babies born with major α-thalassemia, especially in the region where the medical care facilities are lacking. SMRT sequencing is an efficient tool to detect the rare and complex variants of thalassemia in prenatal diagnosis.

SMRT sequencing can not only detect large deletions in one reaction with a pair of primers but also customize the detection of rare variants of thalassemia. In this study, the deletions in subject 1, 3 and 4 were identified using custom designed primers based on the result of MLPA, instead of using universal primers. This means that SMRT sequencing sometimes needs to rely on MLPA result to design primers and amplify for sequencing. The reliance of SMRT sequencing on the MLPA result may, to some extent, be one of its limitations. However, as a large number of new mutations are discovered and accumulated, the primers used to detect these mutations may be used as routine primers for SMRT sequencing after iteration.

In conclusion, this study was novel in identifying four large deletions using SMRT in the α-globin locus in four Chinese individuals. Of these, subject 4 also had a duplication in the deletional region. The findings of this study broadened the spectrum of deletional α-thalassemia and provided a perspective for further studies of the function of the α-globin cluster. Moreover, considering the easily missed diagnosis using conventional methods, SMRT sequencing might be extremely significant for diagnosing rare and complex variants of thalassemia, especially in prenatal diagnosis.
